# Comparative Topographic and Tomographic Assessment of Post‐LASIK Corneal Ectasia

**DOI:** 10.1155/joph/3011668

**Published:** 2026-06-23

**Authors:** Abdelrahman Salman

**Affiliations:** ^1^ Al-Mashrek Laser Vision Center, Tartous, Syria

## Abstract

**Purpose:**

To evaluate and compare the topographic and tomographic characteristics of post–laser in situ keratomileusis (LASIK) ectasia (PLE) with keratoconus (KC) and normal control eyes using the Sirius imaging system.

**Methods:**

This comparative cross‐sectional study included eyes with KC, PLE ectasia, and normal control eyes. All PLE cases developed ectasia following myopic LASIK. Normal control eyes were selected from refractive surgery candidates with normal preoperative corneal tomography and no evidence of ectasia during a postoperative follow‐up period exceeding 1 year. Corneal topography and tomography were performed using the Sirius imaging system. Uncorrected and corrected distance visual acuities were recorded and expressed in decimal notation. Statistical analysis was performed using the Kruskal–Wallis test with post hoc Mann–Whitney U testing. Normality was assessed using the Shapiro–Wilk test. Due to non‐normal distribution, nonparametric tests were used.

**Results:**

A total of 56 eyes were analyzed. KC eyes demonstrated significantly steeper anterior keratometric values compared to PLE and normal control eyes (*p* < 0.001). PLE eyes showed lower anterior keratometric values compared to KC, but remained significantly different from normal controls (*p* < 0.001). Minimum corneal thickness was significantly reduced in both ectatic groups compared to normal controls (*p* < 0.001), with no statistically significant difference between KC and PLE eyes. Posterior tomographic indices were significantly elevated in both ectatic groups compared to normal controls (*p* < 0.001).

**Conclusion:**

PLE demonstrates distinct topographic and tomographic characteristics compared to KC. Comprehensive corneal tomography is essential for accurate evaluation of post‐LASIK ectasia.

## 1. Introduction

Laser in situ keratomileusis (LASIK) has become one of the most widely performed and successful refractive surgical procedures for the correction of ametropia. Despite its high safety profile and predictable outcomes, post‐LASIK ectasia (PLE) remains one of the most serious and potentially vision‐threatening complications [1]. This condition is characterized by progressive biomechanical weakening of the cornea, resulting in structural deformation and refractive instability that may clinically resemble keratoconus (KC), yet develops in a fundamentally different biomechanical and surgical context [2, 3].

The early diagnosis of PLE continues to represent a significant clinical challenge. In corneas that have undergone refractive surgery, conventional anterior surface topography alone may be insufficient to detect subtle structural alterations, particularly in the early stages of ectatic progression [4–7]. Accordingly, this limitation has led to increasing reliance on corneal tomography, which provides three‐dimensional assessment of the anterior and posterior corneal surfaces, corneal thickness measurements, and elevation profiles, allowing for a more comprehensive evaluation of corneal integrity and biomechanical behavior.

Previous studies have extensively described the topographic and tomographic characteristics of primary KC [8–10]. In this context, we have previously investigated corneal tomographic parameters of KC in a population from the Syrian Arab Republic, identifying characteristic elevation and curvature patterns that aid in distinguishing keratoconic from normal corneas [4]. While these findings contributed to improved understanding of primary ectatic disease, extrapolating KC‐based parameters to surgically altered corneas remains problematic, given the distinct biomechanical alterations induced by LASIK.

Despite the clinical importance of PLE, there is a relative paucity of data specifically addressing its tomographic and topographic profile. In particular, the comprehensive characterization of anterior and posterior elevation patterns, curvature asymmetries, and thickness‐related parameters unique to PLE remains limited. Furthermore, direct comparative analyses between PLE, primary KC, and normal corneas using the same imaging platform are scarce.

The purpose of the present study is to characterize the corneal topographic and tomographic parameters of PLE using a combined Scheimpflug–Placido imaging system (Sirius, CSO, Italy) and to compare these parameters with those observed in eyes with KC and in normal control eyes. By identifying distinctive imaging features and quantitative differences among these groups, this study aims to enhance the understanding of PLE, facilitate earlier and more accurate diagnosis, and support more informed clinical decision‐making regarding monitoring and therapeutic intervention in eyes at risk for ectatic progression.

## 2. Materials and Methods

### 2.1. Study Design and Setting

This retrospective study was approved by the Department of Ophthalmology and Research Ethics Committee of Latakia University Hospital, Latakia, Syria (Approval no. LUH‐REC‐2024‐01), and adhered to the tenets of the Declaration of Helsinki. The study involved retrospective analysis of anonymized clinical and corneal imaging data collected during routine ophthalmic practice at Al‐Mashrek Laser Vision Center, Tartous, Syria. Due to the retrospective design of the study and the use of fully de‐identified data, the requirement for written informed consent was waived by the committee.

### 2.2. Study Groups

The study included eyes from three distinct groups: KC, PLE, and normal control eyes (N). All analyses were performed on a per‐eye basis.

In cases where both eyes from the same patient were included, each eye was analyzed independently. No adjustment for intereye correlation was performed due to sample size, and this is acknowledged as a limitation.

KC was diagnosed based on characteristic clinical and tomographic findings, including asymmetric corneal curvature, abnormal anterior and posterior elevation patterns, and localized corneal thinning consistent with ectasia. Device‐derived software indicators provided by the Sirius system were considered supportive but not determinative, and the final diagnosis was established by the examining clinician based on the overall clinical and tomographic assessment [11].

PLE was defined by a history of myopic LASIK exclusively, followed by the development of ectatic changes with compatible Sirius tomography. Diagnostic tomographic features included asymmetric corneal curvature, localized corneal steepening or irregularity with abnormal anterior and/or posterior elevation, and localized corneal thinning, with or without a reduction in corrected distance visual acuity (CDVA) [12].

Where available, the time interval from LASIK to ectasia was reviewed. Preoperative LASIK parameters (ablation depth, flap thickness, stromal bed) were not consistently available due to the retrospective design.

Normal control eyes were recruited from candidates for refractive surgery prior to undergoing LASIK. All tomographic and refractive data for this group were obtained preoperatively. These eyes were not subjected to refractive surgery within the study period. All demonstrated normal corneal tomography without signs of ectasia, a CDVA of 1.0 or better (decimal notation), and remained free of ectatic changes during at least 1 year of clinical follow‐up.

### 2.3. Inclusion and Exclusion Criteria

Inclusion criteria were the availability of high‐quality corneal tomography obtained using the Sirius imaging system, clear classification into one of the three study groups, and complete clinical and tomographic data.

Exclusion criteria included previous corneal surgery other than LASIK (for the PLE group), history of corneal trauma or infection, corneal scarring affecting tomographic analysis, poor‐quality tomography scans, or concurrent ocular pathology affecting corneal shape or visual acuity.

### 2.4. Ophthalmic Examination

All eyes underwent a comprehensive ophthalmic evaluation, including measurement of uncorrected distance visual acuity (UDVA), CDVA, and manifest refraction (sphere and cylinder). Visual acuity was recorded and expressed in decimal notation.

Corneal imaging was performed using the Sirius imaging system (CSO, Italy), which combines a rotating Scheimpflug camera with Placido‐disc topography to provide simultaneous assessment of the anterior and posterior corneal surfaces.

Patients wearing soft contact lenses were instructed to discontinue lens wear for at least 2 weeks prior to tomographic examination, while those wearing rigid gas‐permeable lenses discontinued lens wear for at least 4 weeks before imaging. Image acquisition was performed after ensuring proper patient alignment and fixation. Only scans with acceptable quality scores, as determined by the device software, were included in the analysis. Images affected by poor fixation, blinking, or motion artifacts were excluded.

The following parameters were analyzed.

 Keratometric parameters: flat keratometry (K1), steep keratometry (K2), and average keratometry (K Avg).
 Pachymetric parameter: minimum corneal thickness (ThMin).
 Tomographic indices: symmetry index front (SIf), KC vertex front (KVf), symmetry index back (SIb), and KC vertex back (KVb).

These parameters were evaluated to assess differences among normal eyes, PLE, and KC.

### 2.5. Statistical Analysis

Statistical analysis was performed using SPSS software (IBM SPSS Statistics, Version 26.0; IBM Corp., Armonk, NY, USA). Comparisons among the three groups were performed using the Kruskal–Wallis test. Normality of data distribution was assessed using the Shapiro–Wilk test. As most variables were not normally distributed, nonparametric tests were applied. Results are presented as mean ± standard deviation for consistency with previous literature. When a statistically significant difference was detected, pairwise comparisons were conducted using the Mann–Whitney *U* test. A *p* value ≤ 0.05 was considered statistically significant. All analyses were performed on a per‐eye basis. The number of patients and eyes per patient were recorded when available.

## 3. Results

A total of 56 eyes were included in the analysis: 24 eyes with KC, 12 eyes with PLE, and 20 normal control eyes. The demographic, refractive, and visual characteristics of the study groups are summarized in Table 1.

**TABLE 1:** Demographic, refractive, and visual characteristics.

Parameter	Keratoconus (*n* = 24)	Post‐LASIK ectasia (*n* = 12)	Normal control (*n* = 20)	Overall p	KC vs. PLE	KC vs. normal	PLE vs. normal
Age (years)	24.25 ± 8.00	28.45 ± 3.56	27.60 ± 4.17	0.043	0.034	0.064	0.595
Sphere (D)	−3.01 ± 2.29	−1.54 ± 2.66	−0.50 ± 0.95	< 0.001	0.010	< 0.001	0.367
Cylinder (D)	−4.30 ± 2.98	−0.88 ± 2.07	−0.49 ± 0.30	< 0.001	0.002	< 0.001	0.032
UDVA (decimal)	0.31 ± 0.29	0.24 ± 0.21	0.97 ± 0.09	< 0.001	0.562	< 0.001	< 0.001
CDVA (decimal)	0.49 ± 0.22	0.50 ± 0.28	1.00 ± 0.00	< 0.001	0.946	< 0.001	< 0.001

*Note:* D = diopters; LASIK = laser in situ keratomileusis; KC = keratoconus; *n* = number of eyes. Values are presented as mean ± standard deviation. Data distribution was assessed using the Shapiro–Wilk test. Overall comparisons were performed using the Kruskal–Wallis test. Pairwise comparisons were performed using the Mann–Whitney *U* test. A *p* value ≤ 0.05 was considered statistically significant.

Abbreviations: CDVA, corrected distance visual acuity; PLE, post‐LASIK ectasia; UDVA, uncorrected distance visual acuity.

A statistically significant difference in age was observed among the three groups (Kruskal–Wallis test, *p* = 0.043). KC eyes were significantly younger than PLE eyes (24.25 ± 8.00 vs. 28.45 ± 3.56 years, *p* = 0.034). No significant age differences were observed between KC and N eyes (*p* = 0.064) or between PLE and N eyes (*p* = 0.595).

The mean sphere differed significantly among the three groups (*p* < 0.001). KC eyes demonstrated significantly greater myopia compared to PLE eyes (*p* = 0.010) and N eyes (*p* < 0.001). No statistically significant difference was observed between PLE and N eyes (*p* = 0.367).

Cylinder values also differed significantly across groups (*p* < 0.001). KC eyes showed significantly higher astigmatism compared to PLE eyes (*p* = 0.002) and N eyes (*p* < 0.001). PLE eyes also demonstrated significantly higher cylinder values than N eyes (*p* = 0.032).

UDVA differed significantly among the three groups (*p* < 0.001). UDVA was significantly worse in KC eyes compared to N eyes (*p* < 0.001). No significant difference was observed between KC and PLE eyes (*p* = 0.562). PLE eyes demonstrated significantly poorer UDVA compared to N eyes (*p* < 0.001).

CDVA also showed a significant difference among the three groups (*p* < 0.001). Both KC and PLE eyes had significantly worse CDVA compared to N eyes (*p* < 0.001 for both comparisons). No statistically significant difference was observed between KC and PLE eyes (*p* = 0.946).

Keratometric and pachymetric parameters are summarized in Table 2.

**TABLE 2:** Keratometric and pachymetric parameters.

Parameter	Keratoconus	Post‐LASIK ectasia	Normal controls	Overall p	KC vs. PLE	KC vs. normal	PLE vs. normal
K1 (D)	45.88 ± 2.80	44.05 ± 3.45	42.75 ± 1.05	< 0.001	0.050	< 0.001	0.830
K2 (D)	49.18 ± 2.63	46.12 ± 3.60	43.53 ± 0.98	< 0.001	0.011	< 0.001	0.022
K Avg (D)	47.46 ± 2.58	45.73 ± 5.21	43.13 ± 1.01	< 0.001	0.022	< 0.001	0.179
ThkMin (μm)	441.04 ± 41.19	429.25 ± 55.43	543.90 ± 24.93	< 0.001	0.827	< 0.001	< 0.001

*Note:* K1 = flat keratometry; *D* = diopters; K2 = steep keratometry; K Avg = average keratometry; ThkMin = minimum corneal thickness; μm = micron; LASIK = laser in situ keratomileusis; KC = keratoconus. Values are presented as mean ± standard deviation. Data distribution was assessed using the Shapiro–Wilk test. Overall *p* values were obtained using the Kruskal–Wallis test, and pairwise comparisons were performed using the Mann–Whitney *U* test. A *p* value ≤ 0.05 was considered statistically significant.

Abbreviation: PLE, post‐LASIK ectasia.

Overall comparisons revealed statistically significant differences among the three groups for all evaluated parameters (Kruskal–Wallis test, *p* < 0.001 for all).

Flat keratometry differed significantly among groups (*p* < 0.001). KC eyes demonstrated significantly steeper K1 values compared to N eyes (45.88 ± 2.80 D vs. 42.75 ± 1.05 *D*, *p* < 0.001). A borderline significant difference was observed between KC and PLE eyes (45.88 ± 2.80 D vs. 44.05 ± 3.45 *D*, *p* = 0.050). No statistically significant difference was detected between PLE and N eyes (*p* = 0.830).

Steep keratometry also differed significantly among the three groups (*p* < 0.001). KC eyes exhibited significantly higher K2 values compared to PLE eyes (49.18 ± 2.63 D vs. 46.12 ± 3.60 *D*, *p* = 0.011) and N eyes (43.53 ± 0.98 *D*, *p* < 0.001). PLE eyes demonstrated significantly steeper K2 values compared to N eyes (*p* = 0.022).

K Avg showed a significant difference among the three groups (*p* < 0.001). KC eyes had significantly higher K Avg values compared to PLE eyes (47.46 ± 2.58 D vs. 45.73 ± 5.21 *D*, *p* = 0.022) and N eyes (43.13 ± 1.01 *D*, *p* < 0.001). The difference between PLE and N eyes was not statistically significant (*p* = 0.179).

ThMin differed significantly among groups (*p* < 0.001). Both KC and PLE eyes demonstrated significantly thinner corneas compared to N eyes (*p* < 0.001 for both). No statistically significant difference in ThMin was observed between KC and PLE eyes (*p* = 0.827).

Sirius‐derived tomographic parameters are summarized in Table 3.

**TABLE 3:** Sirius tomographic parameters.

Parameter	Keratoconus	Post‐LASIK ectasia	Normal controls	Overall p	KC vs. PLE	KC vs. normal	PLE vs. normal
SIf (D)	6.61 ± 3.75	4.84 ± 3.78	0.05 ± 0.49	< 0.001	0.159	< 0.001	< 0.001
KVf (μm)	29.08 ± 14.26	23.22 ± 14.31	3.60 ± 1.19	< 0.001	0.491	< 0.001	< 0.001
SIb (D)	1.74 ± 0.73	1.28 ± 0.88	0.00 ± 0.07	< 0.001	0.070	< 0.001	< 0.001
KVb (μm)	64.79 ± 24.72	66.24 ± 41.27	9.35 ± 2.56	< 0.001	0.946	< 0.001	< 0.001

*Note:* SIf, surface asymmetry index front; μm, micron; Sib, surface asymmetry index back; KVb, keratoconus vertex back; LASIK, laser in situ keratomileusis; KC, keratoconus. Values are presented as mean ± standard deviation. Data distribution was assessed using the Shapiro–Wilk test. Overall *p* values were obtained using the Kruskal–Wallis test, and pairwise comparisons were performed using the Mann–Whitney *U* test. A *p* value ≤ 0.05 was considered statistically significant.

Abbreviations: KVf, keratoconus vertex front; PLE, post‐LASIK ectasia.

Statistically significant overall differences were observed among the three groups for all evaluated indices (Kruskal–Wallis test, *p* < 0.001).

SIf differed significantly among groups (*p* < 0.001). Both KC and PLE eyes demonstrated significantly higher SIf values compared to N eyes (*p* < 0.001 for both). The difference between KC and PLE eyes did not reach statistical significance (*p* = 0.159). KVf values showed a significant overall difference (*p* < 0.001). KC eyes exhibited significantly higher anterior vertex elevation compared to N eyes (*p* < 0.001). PLE eyes also demonstrated significantly higher KVf values compared to N eyes (23.22 ± 14.31 vs. 3.60 ± 1.19; *p* < 0.001). Although KVf values were numerically lower in PLE eyes than in KC eyes, this difference was not statistically significant (*p* = 0.491). SIb differed significantly among the three groups (*p* < 0.001). Both KC and PLE eyes showed significantly higher SIb values compared to N eyes (0.00 ± 0.07; *p* < 0.001 for both). The difference between KC and PLE eyes did not reach statistical significance (*p* = 0.070). KVb also demonstrated a significant overall difference among groups (*p* < 0.001). Both KC and PLE eyes exhibited significantly higher KVb values compared to N eyes *p* < 0.001 for both). No statistically significant difference was observed between KC and PLE eyes (*p* = 0.946).

## 4. Discussion

The present study evaluated the topographic and tomographic characteristics of PLE in comparison with KC and normal control eyes using the Sirius imaging system. Although PLE and KC are both classified as corneal ectatic disorders, the results of this study demonstrate that they exhibit distinct tomographic patterns, particularly with respect to anterior keratometric behavior.

Beyond these baseline observations, further clinical implications can be derived from the comparative analysis. Most currently used diagnostic indices and software platforms, including Sirius and Pentacam BAD, have been primarily developed and validated for the detection of KC [13]. However, PLE represents a fundamentally different biomechanical and structural condition [14]. In these eyes, prior myopic ablation induces central corneal flattening, which may mask or alter classical anterior surface indicators typically used in KC detection. Therefore, relying solely on conventional KC‐based diagnostic indices may lead to underdiagnosis or misinterpretation in PLE cases, particularly in early stages. In this context, the present study provides a focused comparative tomographic analysis between KC and PLE, highlighting the need for refined or potentially distinct diagnostic approaches tailored to postrefractive ectasia. These findings may serve as a preliminary step toward the development of more specific diagnostic algorithms for detecting PLE at earlier stages. These findings should be interpreted as an incremental refinement of existing knowledge rather than a major conceptual advance.

From a biomechanical perspective, these differences can be further explored through anterior surface behavior. In the current cohort, KC eyes demonstrated significantly steeper anterior keratometric values compared to PLE eyes. This finding is consistent with the established pathophysiology of KC as a primary ectatic disease characterized by progressive anterior corneal steepening and irregularity [15]. In contrast, PLE eyes demonstrated less pronounced anterior corneal steepening compared to KC, despite clear tomographic evidence of ectasia. This observation can be explained by the biomechanical effects of myopic LASIK surgery, in which central stromal tissue ablation results in intentional flattening of the central anterior cornea [16]. Consequently, ectatic changes developing after LASIK may not manifest as marked central anterior steepening, particularly in the early or moderate stages of the disease. Instead, PLE often originates outside the optical ablation zone, leading to localized or paracentral corneal deformation rather than uniform central curvature elevation [17].

To further contextualize these findings, the underlying mechanisms of PLE should be considered. The development of PLE is generally attributed to biomechanical weakening of the cornea following refractive surgery [8, 9]. The creation of the corneal flap and subsequent stromal ablation reduce the structural integrity of the cornea, particularly when combined with preexisting susceptibility factors such as subclinical KC, reduced corneal thickness, or high ablation depth [20]. In many cases, ectasia represents a combination of iatrogenic weakening and an underlying predisposition rather than a purely surgical complication. This may explain why PLE demonstrates different topographic and tomographic characteristics compared to primary KC, as prior myopic ablation alters the anterior corneal surface and may partially mask early ectatic changes. In the present study, the absence of detailed preoperative data limits the ability to identify specific risk factors in individual cases; however, the observed differences between PLE and KC support the concept that these are biomechanically distinct entities despite sharing similar clinical endpoints.

**FIGURE 1:**
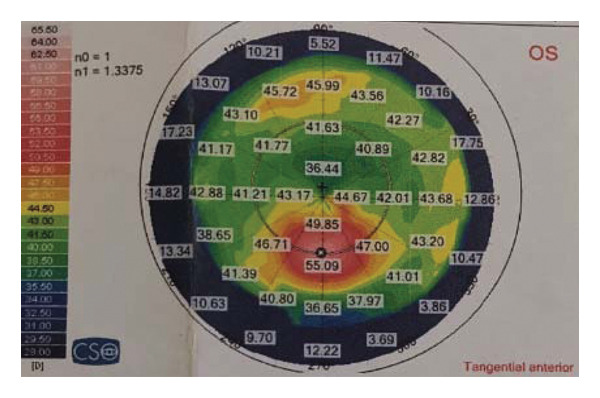
Representative Sirius tomographic map of post‐LASIK ectasia. The tangential anterior curvature map demonstrates localized inferior steepening predominantly outside the central optical zone, with the ectatic apex displaced inferiorly (*r* = 3.4 mm from the corneal center), while the central cornea remains relatively flattened, consistent with prior myopic laser ablation.

This anterior curvature behavior is well illustrated by the representative PLE case shown in Figure 1. The tangential anterior curvature map demonstrates localized inferior steepening predominantly located outside the central optical zone, while the central cornea remains relatively flattened. The displacement of the ectatic apex from the corneal center (*r* = 3.8 mm) further supports the paracentral nature of ectatic involvement in PLE and visually reinforces the quantitative findings of the present study.

**FIGURE 2:**
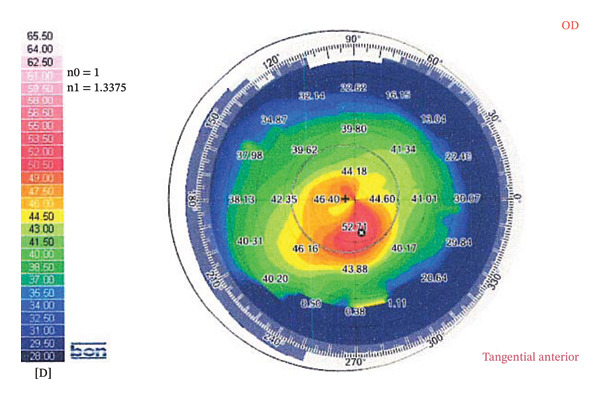
Representative tangential anterior curvature map of a keratoconus eye obtained using the Sirius imaging system. The tangential anterior curvature map demonstrates pronounced central and paracentral corneal steepening corresponding to the cone location, consistent with the characteristic anterior curvature pattern of primary ectatic disease.

For illustrative comparison, a representative tangential anterior curvature map of a KC eye is shown in Figure 2. In contrast to PLE, KC demonstrates pronounced central and paracentral anterior corneal steepening corresponding to the cone location. This anterior curvature pattern reflects the primary nature of KC as a disease dominated by progressive anterior corneal deformation, whereas PLE may exhibit attenuated central anterior steepening due to prior myopic laser ablation.

These observations emphasize that reliance on keratometry or corneal thickness alone may be insufficient when evaluating patients with suspected PLE. Comprehensive corneal tomography provides a more accurate assessment of ectatic changes and improves understanding of the distinct biomechanical behavior of postrefractive surgery corneas. Improved recognition of these tomographic characteristics may facilitate earlier diagnosis.

In addition to anterior findings, posterior corneal parameters also contribute to differentiation. PLE eyes showed significant deviations from normal controls on Sirius tomography, particularly in posterior surface indices, underscoring the diagnostic value of posterior corneal assessment in postrefractive surgery eyes. Our findings are in agreement with those reported by Padmanabhan et al., who evaluated the tomographic characteristics of PLE eyes in comparison with normal controls using corneal imaging [21]. In their study, PLE eyes demonstrated significant deviations from normal corneas, particularly in posterior corneal parameters, highlighting the importance of comprehensive tomographic assessment in postrefractive surgery eyes. However, an important distinction between the two studies should be emphasized. While Padmanabhan et al. focused on differentiating PLE from normal eyes, the present study additionally included a KC group, allowing a more comprehensive comparison between primary and secondary ectatic disorders. This three‐group design enabled us to demonstrate that PLE shares certain ectatic features with KC while exhibiting distinct anterior keratometric characteristics likely related to prior myopic laser ablation.

Accordingly, our results expand upon previous observations by demonstrating that PLE cannot be fully characterized by comparisons with normal corneas alone, and that inclusion of KC eyes provides important insights into the unique biomechanical and tomographic behavior of postrefractive surgery ectasia.

In recent years, there has been increasing interest in topography‐guided excimer laser ablation combined with corneal collagen cross‐linking as a therapeutic approach for selected cases of KC and PLE [22, 23]. Accurate characterization of anterior corneal surface behavior and asymmetry patterns is essential for appropriate treatment planning and customization of such procedures. In this context, the findings of the present study may contribute to improved understanding of anterior corneal alterations in ectatic disorders, thereby supporting more informed decision‐making in contemporary management strategies.

These observations are further supported by previously published literature. Zhao et al. demonstrated that corneal biomechanical properties differ significantly between the two conditions, with KC exhibiting greater biomechanical weakening compared to PLE [24]. Similarly, previous studies suggest that although both conditions share features of ectatic corneal disease, their underlying mechanisms are distinct [20]. These findings support the concept that PLE should not be considered merely a variant of KC, but rather a separate clinical entity with unique diagnostic and biomechanical characteristics.

Despite these findings, several limitations should be considered when interpreting the results. The relatively small sample size of the PLE group reflects the rarity of the condition and may limit generalizability. The retrospective design restricted the availability of important clinical variables, including preoperative refractive data, pachymetry, ablation parameters, and the interval between LASIK and ectasia diagnosis.

In addition, potentially relevant parameters such as intraocular pressure, corneal biomechanical properties (e.g., Ocular Response Analyzer), and corneal densitometry were not available. Some patients contributed both eyes, and no adjustment for intereye correlation was performed. Although appropriate nonparametric tests were used, no correction for multiple comparisons or reporting of effect sizes was included. Finally, diagnostic performance analyses such as ROC curves were not performed, and all measurements were obtained using a single imaging device. Further prospective studies with larger cohorts and more comprehensive datasets are needed.

## 5. Conclusion

PLE demonstrates distinct topographic and tomographic characteristics compared to KC and normal corneas. Comprehensive corneal tomography is essential for accurate diagnosis and improved understanding of PLE. Future studies with larger cohorts are needed to refine early diagnostic criteria and guide optimal management strategies for this condition.

## Author Contributions

Abdelrahman Salman: conceptualization, study design, data collection, statistical analysis, interpretation of results, manuscript drafting, and final approval.

## Funding

This research received no external funding.

## Ethics Statement

This retrospective study was approved by the Department of Ophthalmology and Research Ethics Committee of Latakia University Hospital, Latakia, Syria (Approval no. LUH‐REC‐2024‐01), and adhered to the tenets of the Declaration of Helsinki. The study involved retrospective analysis of anonymized clinical and corneal imaging data collected during routine ophthalmic practice at Al‐Mashrek Laser Vision Center, Tartous, Syria. Given the retrospective nature of the study and the use of fully de‐identified data, the requirement for written informed consent was waived by the committee.

## Consent

The author has nothing to report.

## Conflicts of Interest

The author declares no conflicts of interest.

## Data Availability

The datasets generated and/or analyzed during the current study are available from the corresponding author on reasonable request.
